# Deep learning nomogram for predicting neoadjuvant chemotherapy response in locally advanced gastric cancer patients

**DOI:** 10.1007/s00261-024-04331-7

**Published:** 2024-05-26

**Authors:** Jingjing Zhang, Qiang Zhang, Bo Zhao, Gaofeng Shi

**Affiliations:** 1 Department of Radiology, The Fourth Hospital of Hebei Medical University, Shijiazhuang, People’s Republic of China; 2 Department of Radiation Oncology, The First Hospital of Qinhuangdao, Qinhuangdao, People’s Republic of China; 3 Department of Medical Imaging, The First Hospital of Qinhuangdao, Qinhuangdao, People’s Republic of China

**Keywords:** Locally advanced gastric cancer, Deep learning, Nomogram, Neoadjuvant chemotherapy, Contrast-enhanced computed tomography

## Abstract

**Purpose:**

Developed and validated a deep learning radiomics nomogram using multi-phase contrast-enhanced computed tomography (CECT) images to predict neoadjuvant chemotherapy (NAC) response in locally advanced gastric cancer (LAGC) patients.

**Methods:**

This multi-center study retrospectively included 322 patients diagnosed with gastric cancer from January 2013 to June 2023 at two hospitals. Handcrafted radiomics technique and the EfficientNet V2 neural network were applied to arterial, portal venous, and delayed phase CT images to extract two-dimensional handcrafted and deep learning features. A nomogram model was built by integrating the handcrafted signature, the deep learning signature, with clinical features. Discriminative ability was assessed using the receiver operating characteristics (ROC) curve and the precision-recall (P-R) curve. Model fitting was evaluated using calibration curves, and clinical utility was assessed through decision curve analysis (DCA).

**Results:**

The nomogram exhibited excellent performance. The area under the ROC curve (AUC) was 0.848 [95% confidence interval (CI), 0.793–0.893)], 0.802 (95% CI 0.688–0.889), and 0.751 (95% CI 0.652–0.833) for the training, internal validation, and external validation sets, respectively. The AUCs of the P-R curves were 0.838 (95% CI 0.756–0.895), 0.541 (95% CI 0.329–0.740), and 0.556 (95% CI 0.376–0.722) for the corresponding sets. The nomogram outperformed the clinical model and handcrafted signature across all sets (all *P* < 0.05). The nomogram model demonstrated good calibration and provided greater net benefit within the relevant threshold range compared to other models.

**Conclusion:**

This study created a deep learning nomogram using CECT images and clinical data to predict NAC response in LAGC patients undergoing surgical resection, offering personalized treatment insights.

**Graphical abstract:**

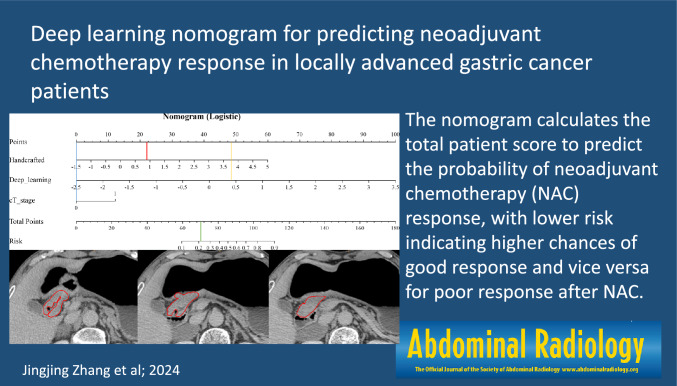

**Supplementary Information:**

The online version contains supplementary material available at 10.1007/s00261-024-04331-7.

## Introduction

Gastric cancer ranks as the fifth most prevalent malignant neoplasm globally and stands as the third most common cause of cancer-related mortality worldwide [1]. Approximately 50% of all gastric cancer cases occur in China [2]. Although surgery serves as the cornerstone of treatment for locally advanced gastric cancer (LAGC), the 5-year survival rate persists at a modest 20–30% [3, 4]. Neoadjuvant chemotherapy (NAC) has become a crucial additional treatment modality for LAGC, aiming to increase the R0 resection rate and improve prognosis by shrinking tumors. Patients with LAGC, stage T2 or above, are recommended to have NAC [5]. However, studies indicate significant individual variability in the response of LAGC patients to NAC, with some developing resistance [6]. For such patients, NAC not only fails to provide additional survival benefits but also introduces unnecessary toxicity and may increase the risk of disease progression [7]. The efficacy of NAC in patients with LAGC can be assessed through methods such as tumor regression grade (TRG) or the Mandard scoring system, which evaluate postoperative tissue [8]. The aforementioned methods evaluate patients’ response to NAC based on the extent of residual tumor in postoperative tissue specimens, providing clinicians with crucial information regarding patients’ treatment response and guiding further treatment strategies. Previous studies have found that for TRG 0 or 1 patients without adverse prognostic factors, the option of no postoperative chemotherapy may be considered, whereas for patients with TRG 2 or 3, postoperative chemotherapy may be necessary [9, 10]. However, these methods rely on pathologic results obtained after tumor surgical resection, making them challenging to apply for predictions before NAC. Currently, tumor staging (Tumor Node Metastasis, TNM) and histological subtype are commonly used for risk stratification and treatment decisions [11]. However, even in patients with similar staging and receiving similar NAC, significant differences in prognosis persist, suggesting that the clinical and pathological risk factors currently employed may not accurately predict the individual patient prognosis after NAC [12]. Therefore, there is an urgent need in clinical practice for biomarkers capable of predicting the response to NAC, providing better guidance for treatment decisions.

Computed Tomography (CT) is presently the predominant imaging modality utilized in clinical settings for the diagnosis, staging, and post-treatment surveillance of gastric cancer. However, conventional CT examinations, based on visual observation, have limited predictive value for NAC prognosis [13]. Radiomics is a data computation and analysis method, based on medical imaging, aiming to uncover features not visible to the naked eye by extracting numerous quantitative features, providing a comprehensive description of tumor heterogeneity [14]. A previous study has shown that image analysis techniques, based on CT radiomics, can offer morphological features, texture, and other characteristics of tumors in CT images, thereby assisting clinicians in predicting the response of gastric cancer patients to NAC [15].

However, radiomics requires manual extraction and selection of features, which may be subject to subjective influences leading to perceived biases. In contrast, deep learning technology, which has been widely utilized across various disciplines, has demonstrated significant advantages [16]. Deep learning models can automatically identify features and representations from raw data through an end-to-end learning process, reducing the need for manual feature extraction. Previous researchers have successfully applied this technology to predict lymph node metastasis, peritoneal metastasis, molecular subtypes, and prognosis of gastric cancer, achieving satisfactory results with good generalization in validation sets [17–19]. Deep learning models exhibit excellent generalization, with features and patterns learned from one dataset potentially adapting well to other datasets. A previous study has shown that the integration of deep learning and handcrafted radiomics features into a nomogram can accurately predict the response of locally advanced cervical cancer patients to NAC, whose performance is significantly superior to that of the clinical model and handcrafted radiomics features [20]. Additionally, some researchers have combined deep learning and handcrafted radiomics for predicting the response of breast cancer to NAC, with its efficacy surpassing that of the clinical model [21]. This study aimed to utilize multi-phase enhanced CT images and leverage both traditional radiomics and deep learning techniques to develop a radiomics clinical features model for prediction of NAC effectiveness. Moreover, this study endeavored to investigate the supplementary value of the model in predicting patients’ progression-free survival (PFS).

## Materials and methods

### Patient and inclusion criteria

The subjects of this study were 414 patients diagnosed with LAGC at two Chinese hospitals. Both institutions had identical inclusion and exclusion criteria. Inclusion criteria included: (a) gastric adenocarcinoma confirmed through histopathological examination; (b) LAGC diagnosis based on preoperative CT scans or laparoscopic examination according to the American Joint Committee on Cancer (AJCC) TNM staging manual (8th edition), defined as cT2 ~ 4/N0 ~ N3/M0; (c) undergoing gastrectomy and lymph node dissection after NAC, with confirmation of TRG on postoperative pathological examination; (d) having undergone multi-phase contrast-enhanced CT scans before treatment. Exclusion criteria included: (a) inability to identify the primary tumor on CT or bad CT image quality (e.g., severe artifacts) preventing accurate measurements; (b) concurrent presence of other malignancies; (c) receiving anticancer treatment before baseline CT scanning; (d) incomplete clinical or pathologic data. Ethical approval was secured from the Ethics Review Committees of both participating medical centers. Given the retrospective nature of the study, informed consent was waived.

**Fig. 1:**
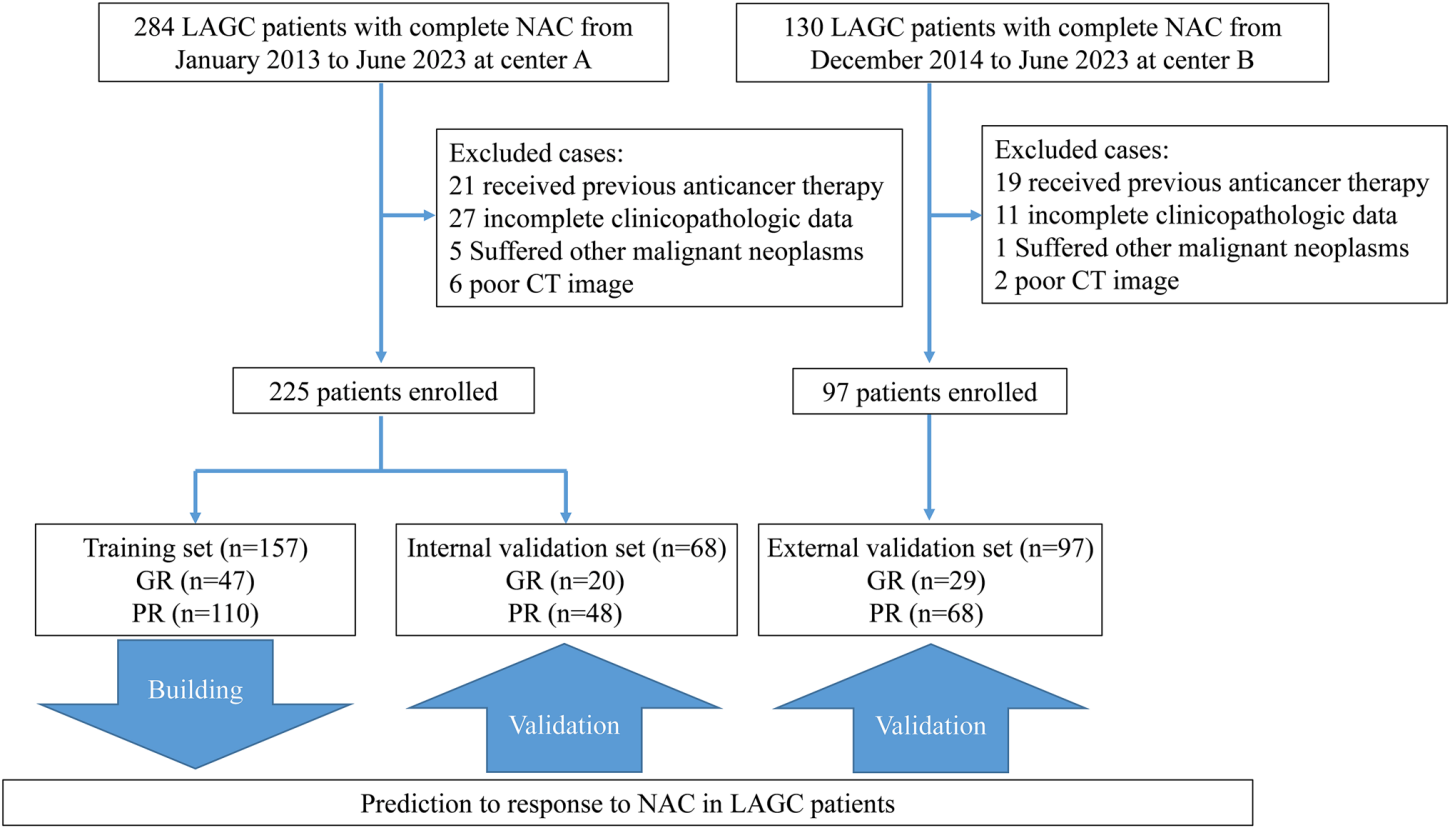
Inclusion and exclusion flowchart for patients in the study. *LAGC* locally advanced gastric cancer, *NAC* neoadjuvant chemotherapy, *CT* computed tomography, *GR* good response, *PR* poor response

This study divided patients into three sets: (1) Training set and internal validation set: A total of 284 LAGC patients who sought medical attention at the Fourth Hospital of Hebei Medical University from January 2013 to June 2023 were initially considered. Following the inclusion/exclusion criteria mentioned above, 225 patients were ultimately included. They were randomly assigned in a 7:3 ratio, resulting in a training cohort (*n* = 157) and an internal validation cohort (*n* = 68). (2) External validation set: A group of 130 patients with LAGC treated at the First Hospital of Qinhuangdao from December 2014 to June 2023 was identified. After applying the same inclusion/exclusion criteria, 97 patients with advanced gastric cancer were ultimately included. For details of patient inclusion and exclusion, refer to Fig. 1.

### Baseline characteristics

Baseline clinical features, including age, gender, body mass index (BMI), tumor differentiation, carcinoembryonic antigen (CEA), carbohydrate antigen 19–9 (CA 19–9), as well as clinical T (cT) and clinical N (cN) staging according to the 8th edition of the AJCC TNM staging system, were extracted from medical records.

### NAC strategy

All enrolled patients underwent 2–4 cycles of neoadjuvant chemotherapy (specifically the SOX regimen: oxaliplatin 130 mg/m^2^ of body surface area administered intravenously on day 1; S-1 administered orally on days 1–14: for individuals with a body surface area less than 1.25m^2^, 40 mg twice daily; for those with a body surface area between 1.25 and 1.5m^2^, 50 mg twice daily; for those with a body surface area greater than 1.5m^2^, 60 mg twice daily) with treatment cycles repeated every 3 weeks. NAC was administered to all patients before surgery, and dose or cycle adjustments were made based on treatment efficacy and patient tolerance. Preoperative treatment efficacy is assessed based on improvement in patient symptoms, normalization or continuous decrease in tumor markers, and reduction in the size of the primary tumor and suspected metastatic lymph nodes observed in CT or magnetic resonance imaging (MRI). All patients received at least two cycles of the SOX regimen, and there were no cases of premature termination of the intended NAC regimen or alterations in the treatment agents. Gastric resection surgery was performed within 2 weeks of completion of NAC.

### NAC response assessment

The assessment of NAC response was conducted collaboratively by two pathology experts with more than 10 years of experience in diagnosing gastrointestinal tumors. Both were blinded to the imaging and clinical data of the patients. The TRG was categorized into four levels based on the most recent National Comprehensive Cancer Network (NCCN) guidelines (2021, version 4, guideline 26), evaluating the extent of tumor regression after preoperative neoadjuvant treatment for gastric cancer: TRG 0: No viable cancer cells (complete response); TRG 1: Residual cancer cells in single or small clusters (moderate response); TRG 2: Residual cancer with fibrosis in the stroma (mild response); TRG 3: Minimal or no tumor regression, with a significant amount of residual cancer cells (poor response). TRG 0 and TRG 1 were combined into the good response (GR) group, while TRG 2 and TRG 3 were categorized as the poor response (PR) group.

### CT examination

After an overnight fast, patients ingested a small amount of warm water and swallowed a gas-producing powder before the examination, followed by immediate CT scanning. The CT scans covered the entire gastric region and axial images were acquired during breath-holding. Contrast-enhanced CT scans in the arterial phase, portal venous phase, and delayed phase were obtained 30, 60, and 180 s, respectively, after injection of contrast agent. The CT image acquisition parameters for Centers A and B are detailed in Supplementary Table S1.

### Image standardization and segmentation

Image standardization in this study involved two steps to reduce data variability between centers. Firstly, all CT images were resampled using cubic spline interpolation to a pixel size of 1 × 1 mm. Secondly, pixel intensities were normalized, transforming the intensity range to − 1024 HU to 1024 HU, and applying a consistent abdominal window with a window level of 50 and a window width of 350.

A radiologist (Radiologist A) with 5 years of experience in diagnosing digestive system tumors manually delineated regions of interest (ROIs) on the three-phase contrast-enhanced images. The segmentation encompassed tumor parenchyma, necrosis, hemorrhage, and cystic areas, resulting in multiple ROIs containing tumor regions for each patient. For each patient, segmentation was performed on arterial, portal venous, and delayed phase images, encompassing all slices standardized to 1 mm thickness containing CT findings of interest. To assess inter-observer consistency, 30 randomly selected patients underwent a re-segmentation process one month after the initial ROI delineation. This time, both Radiologist A and another radiologist (Radiologist B) with 10 years of experience in diagnosing digestive system tumor diagnosis performed the segmentation using the same method. Inter-group correlation coefficients and intra-group correlation coefficients were calculated for feature extraction. The process of image segmentation is illustrated in Supplementary Fig. S1.

### Manual feature extraction

Manual feature extraction was conducted using the PyRadiomics software package, a Python-based tool. The extracted features comprised 8 first-order statistical features, 18 shape features, 75 s-order statistical features, and 1488 transformation features. Detailed definitions of these features are available at http://PyRadiomics.readthedocs.io/en/latest/. Transformations were applied, including wavelet, Laplacian of Gaussian (LoG) filter, square, square root, logarithm, exponential, gradient, and 2D local binary pattern (LBP2D). Image features were extracted at various spatial scales by adjusting the parameter sigma values of the LoG filter to 3.0 and 5.0. In total, 1589 manual features were extracted from each 2D image. The process of feature extraction is depicted in Supplementary Fig. S1.

### Deep learning feature extraction

For all patients, we included all 2D images containing tumor tissue and their corresponding regions of interest (ROIs) as inputs. Subsequently, the image sizes were uniformly transformed to 224 × 224 pixels to match the input size of the model. This operation, allows for a more comprehensive utilization of medical images, contributing to the improvement of the model’s performance as opposed to selecting only the maximum tumor layer as input. Additionally, since effective training of deep learning models involves millions of learnable parameters for estimation, requiring a large amount of image data, and medical image datasets are often limited in size, this study employed transfer learning techniques to address the issue of insufficient image quantity. Specifically, this study employed the EfficientNet V2 architecture of a pre-trained deep learning model trained on the ImageNet dataset. The final fully connected layer was removed to create a feature extractor, and the resulting output values from the feature extractor were utilized as deep learning features. The images underwent three transformations based on the normalization parameters (z-score) of the red/green/blue channels to adapt them to the format suitable for the ImageNet dataset. Implementation of the EfficientNet V2 model was carried out using the Python timm package (https://github.com/huggingface/pytorch-image-models) in conjunction with the PyTorch library (https://www.pytorch.org/). A total of 1280 features were extracted for each 2D image.

During model training, a cross-entropy loss function was used, and optimization was performed using the Adam algorithm. A batch size of 64, an initial learning rate of 0.1, and a learning rate scheduler (ReduceLROnPlateau) dynamically adjusting the learning rate were employed for training over 100 epochs. The training process was implemented using PyTorch in an environment with an NVIDIA GTX 3090 GPU, Intel(R) Core(TM) CPU i7-12700F @ 2.10, and 64 GB of memory. The feature extraction process is illustrated in Supplementary Fig. S1.

### Feature selection and signature building

To deal with the imbalance between PR and GR samples, we use the borderline synthetic minority over-sampling technique (Borderline-SMOTE) to oversample the training set, ensuring an equal number of GR and PR samples. Then, the handcrafted and deep learning features from the arterial, portal venous, and delayed phases were combined for feature selection and signature building. Firstly, the z-score method was applied to normalize the handcrafted and deep learning features.

The feature selection in this study comprised five steps. Firstly, the ICC value was calculated for both intra-observer and inter-observer reliability. Features with ICC values > 0.75 were considered to have good repeatability and were selected for further filtering. Subsequently, the Spearman correlation coefficient method was used to select features, randomly removing one of two features if their correlation coefficient exceeded 0.9. Next, the variance threshold method was employed to further filter the remaining features. The basic idea was to eliminate features with low variance, as these features exhibit minimal changes in the data and may contribute less information to modeling or classification tasks. In this study, a variance threshold of 0.8 was set; as commonly employed in previous studies [22]. Following that, the ReliefF algorithm, which calculates importance scores by considering the distribution of instance weights based on nearest neighbors, was applied for feature selection. With n_neighbors set to 10 in this study, the ReliefF algorithm selected 10 nearest neighbors of the same class and 10 nearest neighbors of a different class for each sample to estimate the importance scores of features. Finally, the Least Absolute Shrinkage and Selection Operator (LASSO) algorithm was employed. Through ten-fold cross-validation, the penalty parameter was optimized to select the most useful predictive features with non-zero coefficients. The feature selection process is illustrated in Supplementary Fig. S1.

For each gastric cancer patient, as there could be multiple layers of CT images and different layers might yield different predictive results, a voting method was used to determine the final classification. By combining the finally selected features and multiplying them by their normalized coefficients, a multivariate logistic regression model was used to calculate the feature identifiers predicting GR. The final outcome comprised two feature signature; handcrafted feature and deep learning feature signature.

#### Establishment of the deep learning radiomics nomogram

In the training set, univariate analysis was conducted to select clinical-pathologic variables with statistical significance (*P* < 0.05). Subsequently, multivariate logistic regression was performed to integrate the three-phase handcrafted, the deep learning signatures, and significant clinical-pathologic factors, constructing a fused nomogram model. This model was compared with clinical models, handcrafted, and deep learning signatures.

Receiver operating characteristic (ROC) curve analysis was applied to measure the discriminative performance of each model. The Delong test was used to compare the discriminative abilities of different models. Calibration curve analysis and the Hosmer–Lemeshow test were employed to evaluate the goodness-of-fit of the models. Net Reclassification Improvement (NRI) and Integrated Discrimination Improvement (IDI) were calculated to compare the performance differences between the fused model and the clinical model. Decision Curve Analysis (DCA) assessed the clinical utility of the models. Kaplan–Meier curves were used to evaluate the association between the radiomics nomogram score and PFS.

#### PFS

Patients in the follow-up set (*n* = 147) underwent follow-up every 3–6 months in the first 2 years’ post-surgery, followed by annual follow-ups. The follow-up duration extended from the time of surgery until March 2023, collecting information on PFS up to the last follow-up. PFS was defined as the duration from the commencement of tumor NAC to the onset of any type of tumor progression or mortality from any cause. All occurrences of disease progression, encompassing both local recurrence and distant metastasis, were evaluated through clinical examination and imaging modalities such as CT, magnetic resonance imaging, or positron emission tomography-computed tomography scans.

#### Statistical analysis

Differences in clinical characteristics among various groups or cohorts were compared using independent *t*-tests or Mann–Whitney U tests for continuous variables. For categorical variables, Fisher’s exact test or the chi-square test was employed as appropriate. The Akaike Information Criterion (AIC) was served as the stopping criterion for the backward stepwise process aimed at determining the optimal feature combination. Kaplan–Meier survival analysis and log-rank tests were employed to assess the probability of PFS. The optimal cutoff values were determined using X-tile software, and patients were stratified into high-risk and low-risk groups. Univariate and multivariate analyses utilizing Cox proportional hazards regression, with backward stepwise elimination and AIC, were conducted to construct the PFS prediction model. All statistical analyses were carried out using R software (version 3.6.3, http://www.R-project.org). Two-sided *P* value less than 0.05 were considered statistically significant.

## Results

### Patient characteristics

The baseline characteristics of 322 patients with LAGC are presented in Table 1. The efficacy of NAC demonstrated a balanced performance across the three sets, with pathologic GR rates of 29.9, 29.4, and 29.9% in the training, internal validation, and external validation sets, respectively. Clinical and pathologic features, including age, gender, pre-NAC CEA, tumor location, and cN stage, showed no significant differences (*P* > 0.05). Significant differences were observed in BMI, tumor differentiation, pre-NAC carbohydrate CA19-9, and cT stage. Additionally, there was no significant difference in the time from CT examination to the initiation of NAC among the three sets (*P* = 0.051).

**Table 1:** Clinical and pathological characteristics of LAGC patients in the training, internal validation, and external validation sets

Characteristics	Training set	Internal validation set	External validation set	*P*
Age (year, mean ± SD)	61.3 ± 9.7	58.3 ± 10.1	61.1 ± 10.1	0.100
BMI (year, mean ± SD)	22.2 ± 3.2	21.6 ± 3.3	23.2 ± 3.4	0.006
Sex (%)				0.794
Female	49 (31.2%)	23 (33.8%)	28 (28.9%)	
Male	108 (68.8%)	45 (66.2%)	69 (71.1%)	
Differentiation				0.021
Well	2(1.3%)	6(8.8%)	8 (8.2%)	
Moderately	40 (25.5%)	20 (29.4%)	18 (18.6%)	
Poorly	115 (73.2%)	42 (61.8%)	71 (73.2%)	
Pre-NAC CEA(%)				0.798
Normal (≤ 5 ng/mL)	75 (47.8%)	35 (51.5%)	50 (51.5%)	
Abnormal (> 5 ng/mL)	82 (52.2%)	33 (48.5%)	47 (48.5%)	
Pre-NAC CA 19–9(%)				0.025
Normal (≤ 20 U/mL)	73 (46.5%)	45 (66.2%)	52 (53.6%)	
Abnormal(> 20 U/mL)	84 (53.5%)	23 (33.8%)	45 (46.4%)	
Locations (%)				0.926
Cardia/fundus	62 (39.5%)	26 (38.2%)	32 (33.0%)	
Gastric body	21 (13.4%)	8 (11.8%)	16 (16.5%)	
Gastric antrum	66 (42.0%)	30 (44.1%)	42 (43.3%)	
Whole stomach	8 (5.1%)	4 (5.9%)	7 (7.2%)	
cT stage				0.009
T2	6 (3.8%)	5 (7.4%)	0 (0%)	
T3	71 (45.2%)	23 (33.8%)	40 (41.2%)	
T4a	71 (45.2%)	34 (50.0%)	40 (41.2%)	
T4b	9 (5.7%)	6 (8.8%)	17 (17.5%)	
cN stage				0.194
N0	27 (17.2%)	5 (7.4%)	12 (12.4%)	
N1	54 (34.4%)	27 (39.7%)	38 (39.2%)	
N2	46 (29.3%)	27 (39.7%)	36 (37.1%)	
N3	30 (19.1%)	9 (13.2%)	11 (11.3%)	
Time from CT to initiation of NAC (day, mean ± SD)	7.3 ± 2.7	7.0 ± 3.6	6.5 ± 2.3	0.051

*BMI* body mass index, *CA 19–9* carbohydrate antigen 19–9, *CEA* carcinoembryonic antigen, *CT* computed tomography, *LAGC* locally advanced gastric cancer, *NAC* neoadjuvant chemotherapy, *SD* standard deviation

### Handcrafted and deep learning signature construction

The mean and standard deviation of the number of ROIs obtained per individual patient were approximately 30.4 ± 14.9. The feature selection process in this study involved a total of 5 steps. Due to the extraction of 1589 handcrafted features and 1280 deep learning features from each of the arterial phase, venous phase, and delayed phase 2D images, a total of 4767 (1589 × 3) handcrafted features and 3840 (1280 × 3) deep learning features were extracted for the same layer. Of these, 2781 and 2196 features were found to have high reproducibility and stability (ICC > 0.75), respectively. After excluding features with strong correlations using the Spearman method, 2268 and 1755 features with weaker correlations were identified. Following the variance threshold method to remove features with low variance, 1370 and 1081 features were retained. The ReliefF method was then employed to include the 100 features most strongly correlated with the intercept variable for each set. Finally, LASSO regression analysis was applied, resulting in 9 and 3 features remaining to construct the handcrafted and deep learning signatures, respectively. The selected features for constructing the handcrafted and deep learning signatures are listed in Supplementary Table S2.

### Performance of handcrafted and deep learning signature models

**Fig. 2:**
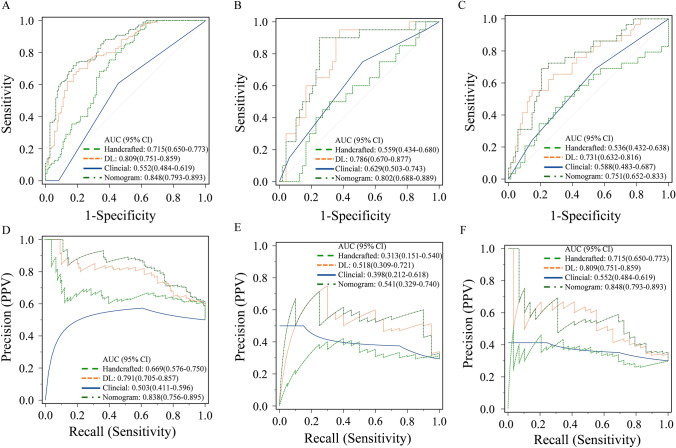
Performance of the four predictive models. **A–C** Receiver operating characteristic curves of three predictive models in** A** the training set, **B** the internal validation set and **C** the external validation set. **D–F** Precision-recall curves of the predictive models in **D** the training set, **E** the internal validation set and **F** the external validation set. *AUC* area under the curve, *DL* deep learning, *PPV* positive predictive value

As shown in Supplementary Table S3, the AUCs of the handcrafted signature in the training, internal validation, and external validation sets were 0.715 (95% CI 0.650–0.773), 0.559 (95% CI 0.434–0.680), and 0.536 (95% CI 0.432–0.638), respectively. The AUCs of the deep learning signature in the training, internal validation, and external validation sets were 0.809 (95% CI 0.751–0.859), 0.786 (95% CI 0.670–0.877), and 0.731 (95% CI 0.632–0.816), respectively. The deep learning signature demonstrated significantly better performance than the handcrafted signature in the training and internal validation sets (with *P* values of 0.028 and 0.010, respectively) but showed no significant difference in the external validation set (*P* = 0.051, Fig. 2 and Supplementary Figure S2).

Furthermore, due to the significant imbalance in the sample sizes between the good response and poor response groups, we also plotted P-R curves to assess the models’ discriminative performance. Compared to the handcrafted feature signature, the deep learning feature signature exhibited a larger area under the P-R curve, and the difference was statistically significant (Supplementary Table S4). The F1_max_ values of the deep learning signature in the training, internal validation, and external validation sets were 0.755, 0.667, and 0.582, respectively (Supplementary Table S3 and Fig. 2).

### Construction of the nomogram

**Fig. 3:**
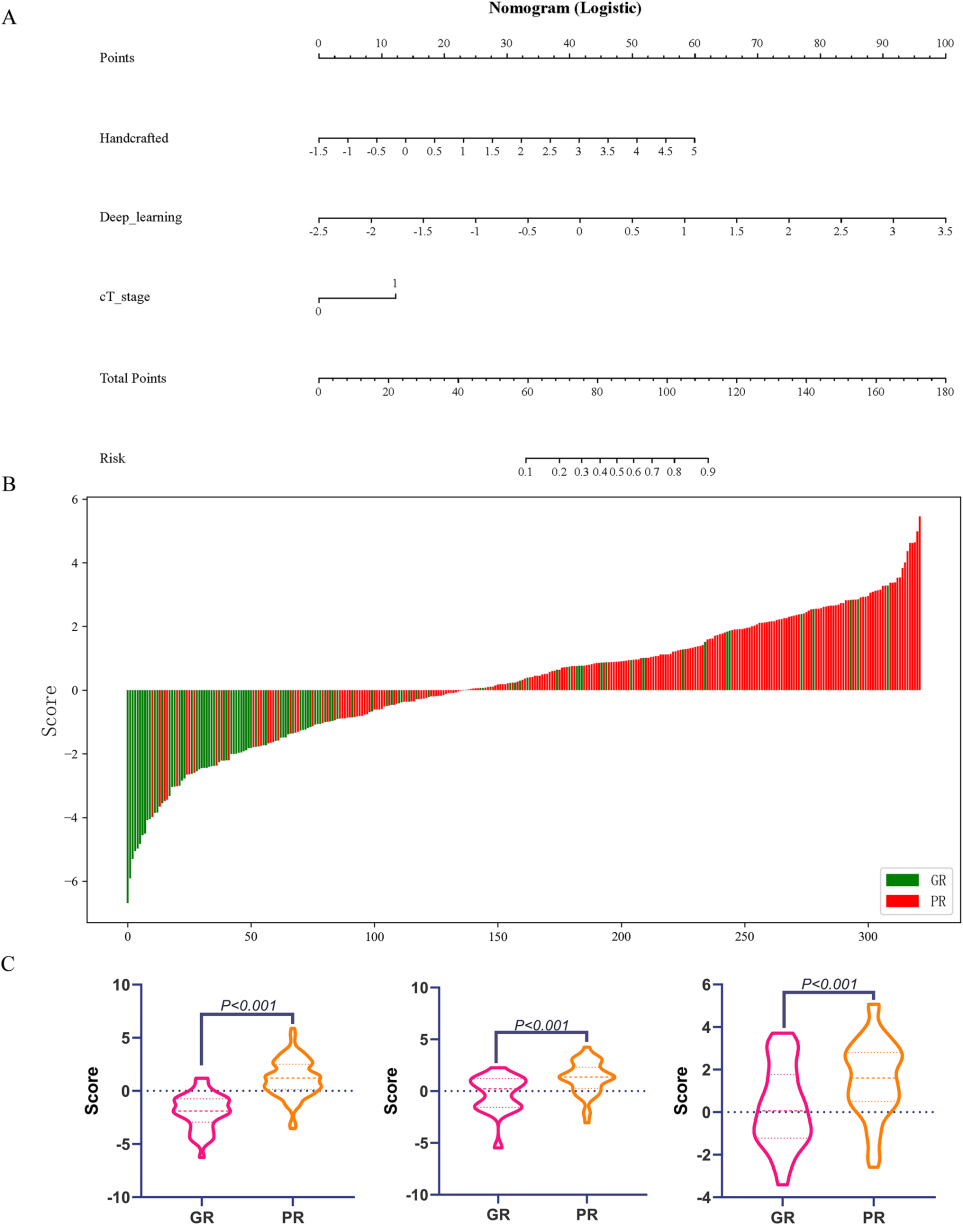
Construction and performance of a combined nomogram model. **A** Nomogram composed of deep learning signature, handcrafted signature, and clinical T stage. For clinical T stage, a score of “0” in the nomogram represents clinical stage T2 or T3, while a score of “1” represents clinical stage T4a or T4b.** B** Waterfall plot illustrating the distribution of nomogram scores for individual cases based on neoadjuvant chemotherapy (NAC) efficacy. **C–E** Violin plots depicting the relationship between nomogram scores and NAC response in the training set **(C)**, internal validation set **(D)** and external validation set **(E)**. *GR* good response; PR: poor response

In the training set, a stepwise backward multivariate analysis using the AIC criterion was performed, and both handcrafted features and cT stage independently emerged as predictors of PR, as detailed in Table S5 and Figure S3. These factors were integrated into the nomogram (Fig. 3A). The AIC of the nomogram model was 213.1, which was lower than the clinical model, handcrafted signature, and deep learning signature (with AICs of 303.7, 270.3, and 235.1, respectively).

**Fig. 4:**
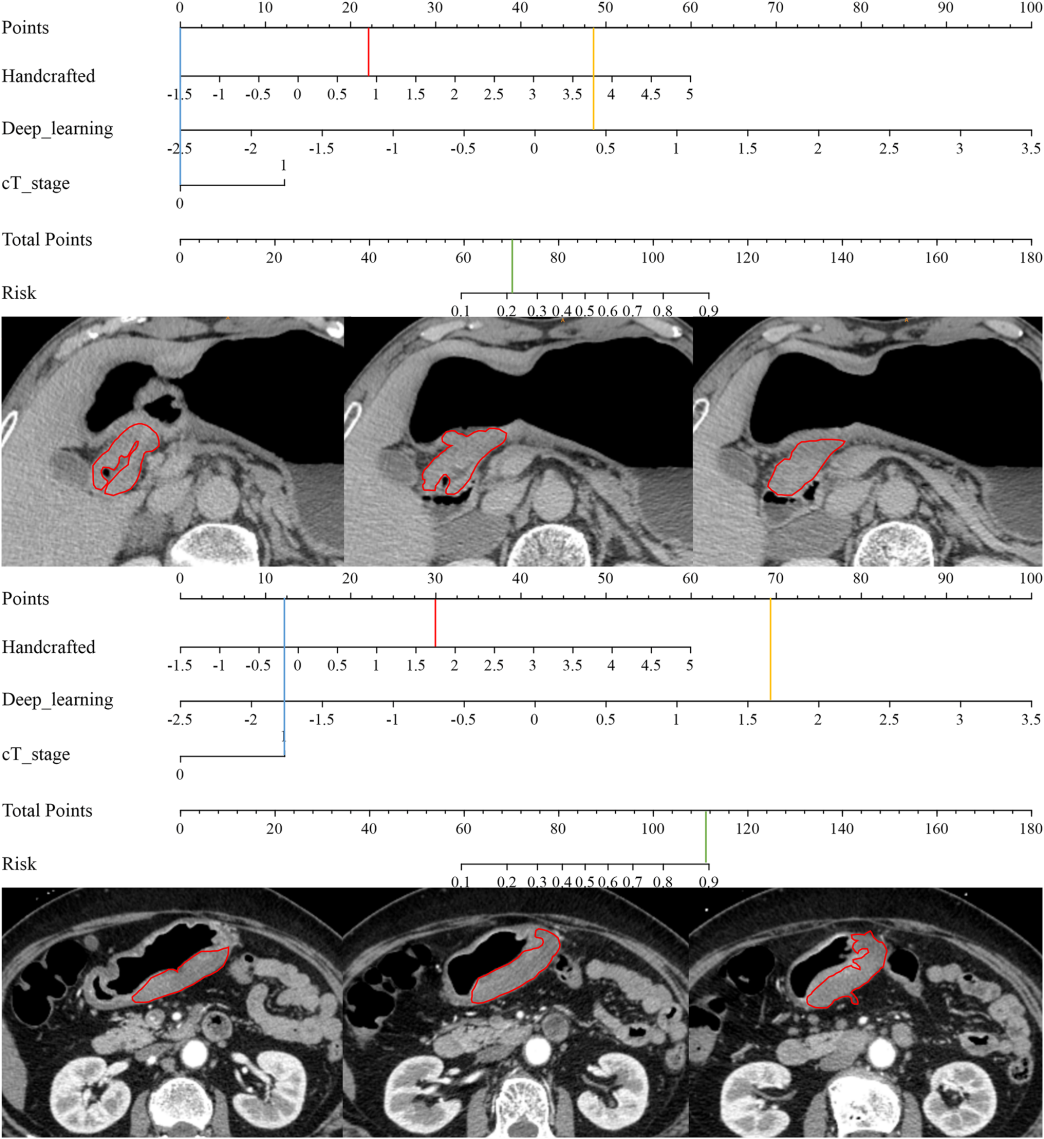
Examples of predicting individual risk for neoadjuvant chemotherapy (NAC) using the nomogram proposed in this study involves three steps. Firstly, vertical lines are drawn for each patient’s variables, where the red, yellow, and blue lines represent handcrafted score, deep learning score, and clinical T stage, respectively. For clinical T stage, a score of “0” in the nomogram represents clinical stage T2 or T3, while a score of “1” represents clinical stage T4a or T4b. Next, the values intersecting each line with the “Points” scale are summed to obtain the total score, referred to as “Total Points”. Finally, a vertical green line is drawn on the “Total points” scale to read the “Risk” of NAC response. **A** The nomogram of a 74-year-old female patient with locally advanced gastric cancer (LAGC) in the gastric antrum. **B–D** The portal venous phase CT images at different slices of the female patient. The female patient’s clinical T stage is 2, with a handcrafted score of 0.812 and a deep learning score of 0.391. By summing the values intersecting the lines with the “Points” scale, the total score is calculated as 0 + 22 + 48 = 70. Drawing a vertical line on the “Total points” scale reveals an approximately 22% risk of poor NAC response for this patient. The patient’s pathological tumor regression grade (TGR) grading after NAC is grade 1. **E** The nomogram of an 52-year-old male patient with LAGC in the LAGC in the gastric body. **F–H** The portal venous phase CT images at different slices of the male patient. The patient’s clinical T stage is 4a, with a handcrafted score of 1.735 and a deep learning score of 1.651. By summing the values intersecting the lines with the “Points” scale, the total score is calculated as 12 + 30 + 69 = 111. Drawing a vertical line on the “Total points” scale reveals an approximately 89% risk of poor NAC response for this patient. The patient’s pathological tumor regression grade (TGR) after NAC is 3

As depicted in Figs. 3C–E, the nomogram scores exhibited significant differences between the GR and PR groups in all datasets (all *P* < 0.001). Risk scores for all patients in the training, internal validation, and external validation sets were computed to visually demonstrate the predictive ability of the model (Fig. 3B). The nomogram model exhibited good performance in the training set and demonstrated robust generalization in the validation sets, with ROC AUCs approximately 0.848 (0.793–0.893), 0.802 (0.688–0.889), and 0.751 (0.652–0.833), and P-R AUCs approximately 0.838 (0.756–0.895), 0.541 (0.329–0.740), and 0.556 (0.376–0.722), respectively. Additionally, the AUCs of the nomogram were significantly higher than that of the clinical model and handcrafted signature in all sets (*P* < 0.05) (Table S3, Fig. 2 and Supplementary Fig. S2). The NRI and IDI analyses indicated that integrating image signatures into the combined nomogram model improved the predictive accuracy of the clinical model across all sets (Table S6). The nomogram demonstrated significantly better performance than the handcrafted signature in the training set (*P* < 0.001), but showed no significant difference in the internal and external validation sets (with *P* values of 0.440 and 0.490, respectively). Examples of using the nomogram to predict the NAC response were presented in Fig. 4.

**Fig. 5:**
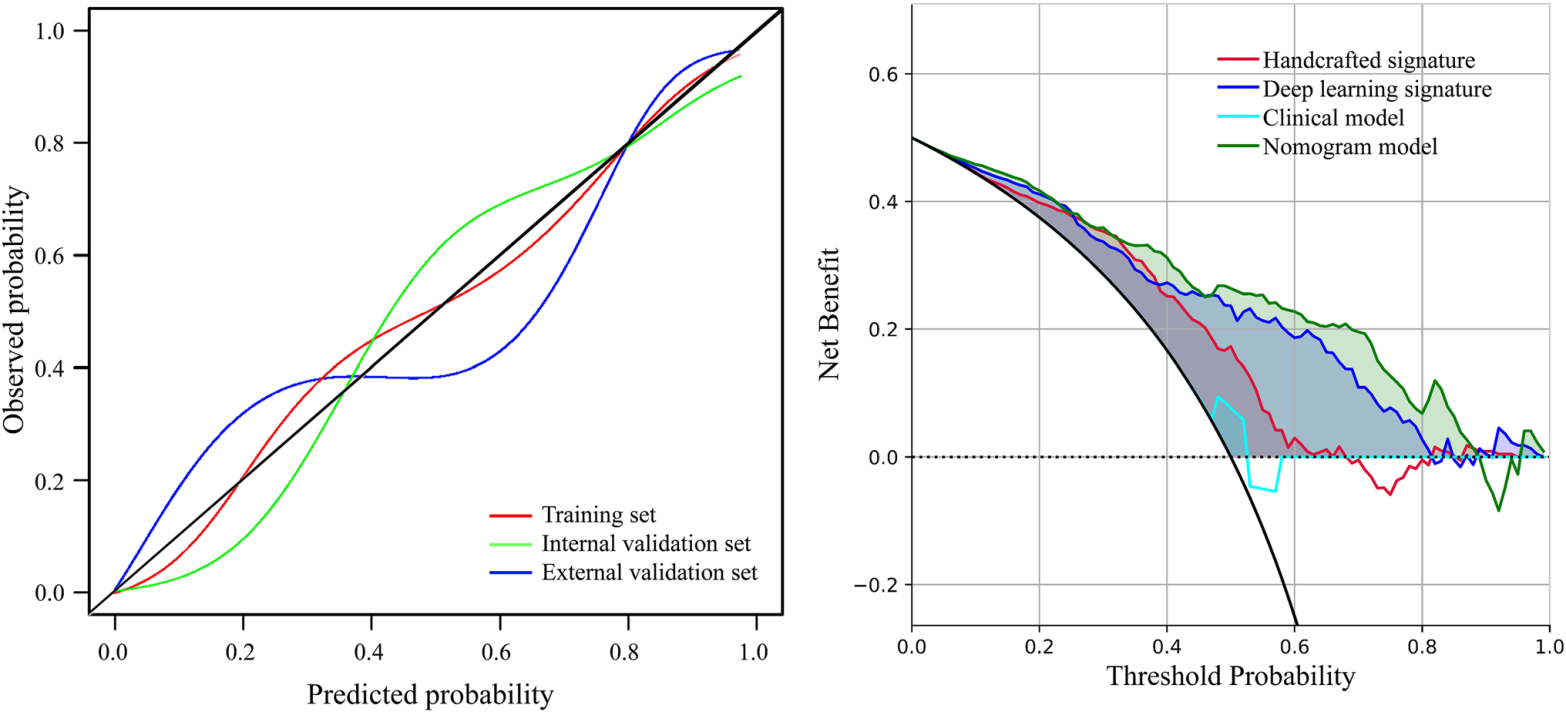
Calibration curves and decision curve analysis (DCA). **A** Calibration curves of combined nomogram model in all the three sets. **B** DCA for handcrafted signature, deep learning signature, clinical model and nomogram model

The calibration curves of the nomogram demonstrated good fitting accuracy between the predicted and observed PR across all cohorts (*P* values for the training, internal validation, and external validation sets were 0.054, 0.430, and 0.441, respectively) (Fig. 5A). Additionally, DCA revealed that the nomogram provided greater net benefit across almost the entire threshold range from 0 to 1 compared to other models across the entire cohort (Fig. 5B). Our radiomics model exhibited higher net benefit almost throughout the entire threshold probability compared to the clinical and other models, enhancing the clinical utility of radiomics in providing recommendations for NAC to patients.

### Predictive factors for PFS in LAGC

**Fig. 6:**
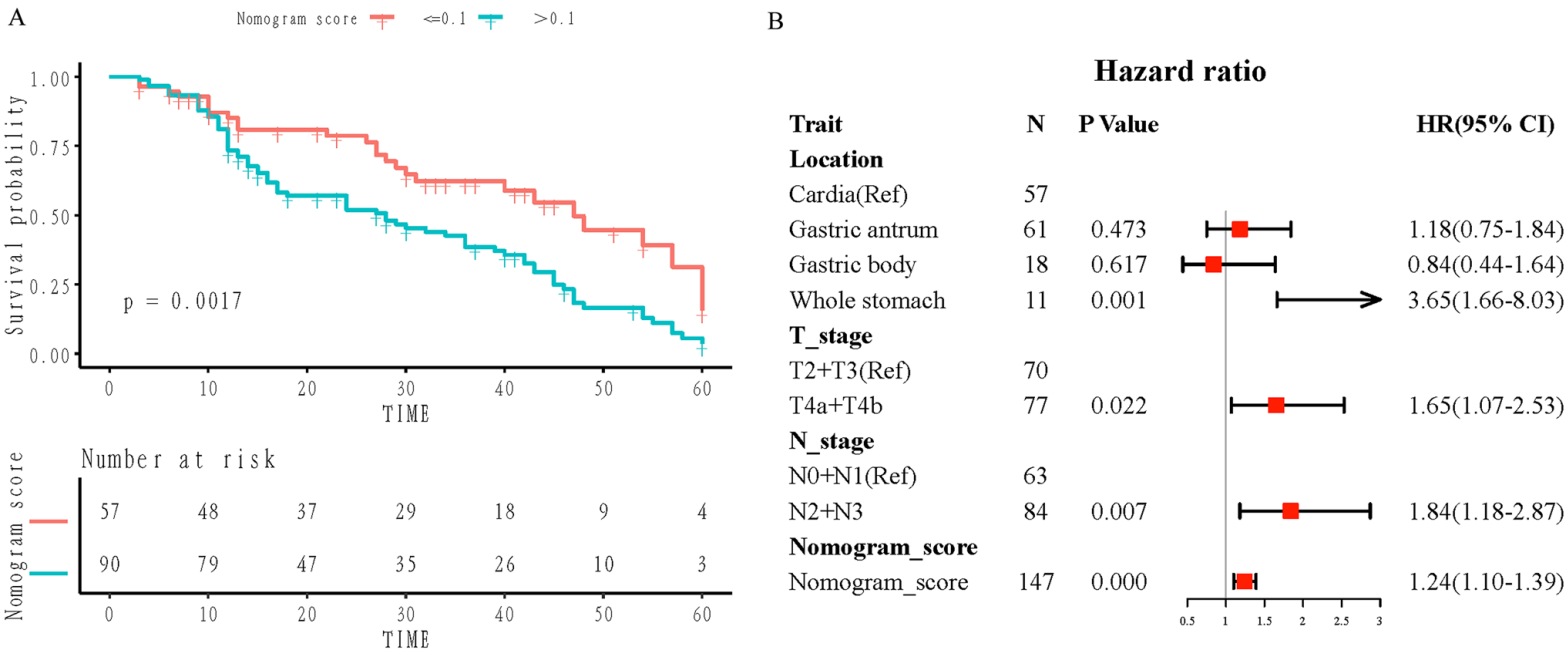
Kaplan–Meier curves and forest plot of progression-free survival (PFS) on the complete cohort. **A** Kaplan–Meier curves of PFS between the groups with low and high signature scores in the complete cohort. **B** Forest plot illustrating multivariable Cox regression analyses for PFS in the complete cohort

We further assessed the prognostic value of the nomogram for patients with LAGC. The median follow-up time and average follow-up durations were 27 and 27.6 months, respectively, with a range of 1 to 65 months. Schoenfeld’s individual test indicated that the Cox model met the proportional hazards assumption (*P* > 0.05). We determined the optimal nomogram score for predicting PFS as 0.1 and subsequently stratified patients into high-risk and low-risk groups using this threshold. The Kaplan–Meier curves demonstrated a significant association between higher nomogram scores and worse PFS (HR = 1.130; 95% CI = 1.016–1.257, *P* = 0.0017) (Fig. 6A). Table 2 presents the findings of univariate and multivariable Cox regression analyses for PFS prediction factors in the follow-up cohort, demonstrating that the nomogram score is an independent prognostic factor for PFS (HR = 1.235; 95% CI = 1.099–1.387, *P* < 0.001), along with pre-NAC full stomach tumor, T stage, and N stage (Fig. 6B). The Cox regression model demonstrated a C-index of 0.673 (95% CI = 0.545–0.802).

**Table 2:** Univariate and multivariate analysis of predictors of PFS

Characteristics	Univariable analysis		Multivariable analysis	
	Hazard ratio (95% CI)	*P*	Hazard ratio (95% CI)	*P*
Age	0.993 (0.973, 1.015)	0.542		
BMI	1.000 (0.938, 1.067)	0.993		
Sex				
Female	Ref			
Male	1.325 (0.859, 2.043)	0.203		
Differentiation				
Well	Ref			
Moderately	0.738 (0.303, 1.803)	0.506		
Poorly	0.897 (0.387, 2.083)	0.801		
Pre-NAC CEA				
Normal	Ref			
Abnormal	0.930 (0.626, 1.382)	0.719		
Pre-NAC CA 19–9				
Normal	Ref			
Abnormal	0.966 (0.646, 1.444)	0.865		
Locations				
Cardia/fundus	Ref		Ref	
Gastric body	0.852 (0.442, 1.640)	0.631	0.843 (0.434, 1.638)	0.615
Gastric antrum	1.143 (0.732, 1.785)	0.557	1.188 (0.761, 1.857)	0.448
Whole stomach	2.084 (1.000, 4.342)	0.050	3.603 (1.639, 7.922)	0.001
cT stage				
T2 + T3	Ref		Ref	
T4a + T4b	1.717 (1.136, 2.594)	0.010	1.642 (1.070, 2.520)	0.023
cN stage				
N0 + N1	Ref		Ref	
N2 + N3	1.779 (1.168, 2.709)	0.007	1.803 (1.159, 2.806)	0.009
Nomogram score	1.126 (1.012, 1.252)	0.029	1.235 (1.099, 1.387)	< 0.001

*95% CI* 95% confidence interval, *BMI* body mass index, *CA 19–9* carbohydrate antigen 19–9, *CEA* carcinoembryonic antigen, *LAGC* locally advanced gastric cancer, *NAC* neoadjuvant chemotherapy, *PFS* progression-free survival

## Discussion

This study developed and validated an innovative machine learning nomogram model that integrates traditional handcrafted radiomics and deep learning features to predict the response to NAC in patients with LAGC undergoing surgical resection. In comparison to clinical and traditional radiomics models, this model exhibited more accurate discriminative ability in the training, internal validation, and external validation sets. Therefore, it processes potential clinical utility in quantifying the risk of adverse individual responses to NAC.

Previous studies have indicated that NAC helps reduce tumor volume, enhances the success rate of surgery [23, 24]. However, a considerable proportion of patients may not derive significant benefits from NAC [25]. Accurately identifying patients who are expected to derive benefit from NAC may contribute to improving its effectiveness. Additionally, a previous study has shown that approximately one-third of LAGC patients may fail to undergo surgical resection after NAC due to poor treatment response or disease progression [26]. For such patients, early identification using a non-invasive approach prior to treatment may aid in selecting more appropriate treatment modalities, thereby improving their prognosis. However, the predictive capability of conventional contrast-enhanced CT examinations for NAC response is limited, making it challenging for clinicians to determine potential responses in patients with LAGC. Radiomics methods can extract a plethora of hidden features from medical images, providing information about tumor microenvironments such as cell density, hypoxia, and microvessel density, significantly expanding the scope of research in medical imaging [27]. Previous researchers have found that traditional handcrafted radiomics signatures can predict the prognosis and NAC response in LAGC patients [28]. A model built with radiomics using multiple machine learning algorithms have shown good discriminative ability in predicting the effectiveness of NAC in gastric cancer, with AUCs of 0.784 and 0.803 in two validation sets [29]. In the study of Zhou et al., handcrafted features were extracted from CT images of 323 gastric cancer patients, demonstrated the discriminative ability of radiomics signatures for NAC response in an external cohort, with an AUC of only 0.679 [15]. Furthermore, the standardization of radiomics features is likely one of the key challenges influencing its widespread application, as there is a lack of reproducibility of radiomics features across different imaging devices and radiomics software. Researchers are continuously striving to address this issue, with initiatives like the Image Biomarker Standardization Initiative (IBSI) proposed by Zwanenburg et al. and undergoing ongoing updates [30]. However, some researchers still argue that IBSI compliance does not guarantee consistency in feature values [31]. In the present study, the performance of the handcrafted signature was poorer, with ROC AUCs of 0.559 and 0.536 for the two validation sets. The reason for this may be that manual feature design and extraction are typically based on prior knowledge and experience. This approach may sometimes fail to adequately capture complex patterns and information in the data, resulting in poor model performance.

To enhance the effectiveness of the handcrafted signature and address its inadequate generalization capability, the emphasis of this study was on examining the contribution of deep learning features. In our research, the deep learning signature showed promising performance, with an ROC AUC exceeding 0.73. This performance is comparable to deep learning features previously employed to predict treatment response in esophageal squamous cell carcinoma [32]. In comparison with conventional radiomics and clinical models, the deep learning signature demonstrated greater predictive efficacy and better generalization. The ROC and PR curve AUCs were significantly higher. Currently, deep learning has transformed the landscape of medical imaging applications. Previous researchers have successfully utilized deep learning techniques to predict lymph node metastasis, depth of infiltration, and survival outcomes in gastric cancer patients, achieving notable results [18, 33]. In the context of predicting response to NAC, researchers previously employed deep learning features extracted from a ResNet-50 network and observed AUC values ranging from 0.752 to 0.808 in the validation sets [34], which aligns closely with the findings of this study. However, that study only extracted features from portal venous phase images, potentially overlooking crucial information present in arterial and delayed phase images. Some researchers have noted a close association between DL features extracted from arterial phase images and the heterogeneity of gastric cancer [19]. Another study utilizing features extracted from a Densenet-121 network accurately predicted NAC response in gastric cancer, achieving AUC values ranging between 0.720 and 0.806 [35]. However, this study only included the maximum tumor layer for each patient, potentially limiting the number of included images. In contrast, our study included all images containing tumors and their corresponding ROCs, greatly enriching the sample size and potentially yielding more accurate predictions. All these findings indicate that deep learning provides rich information reflecting tumor spatial heterogeneity and the relationship between the tumor microenvironment and chemotherapy sensitivity.

In our study, we employed the recently introduced, lightweight deep learning network known as EfficientNet V2 [36]. EfficientNet V2 represents an upgraded version of the deep neural network model developed by Google, with the primary goal of further enhancing model efficiency and performance. In comparison to the original EfficientNet, EfficientNet V2 incorporates more efficient architectural designs and optimization strategies, achieving improved performance while maintaining a relatively smaller model size. It combines optimizations in model depth, width, and resolution to provide enhanced accuracy, particularly in scenarios with limited computational resources [37].

Previous studies have indicated that clinical-pathologic features, including age, tumor stage, and tumor differentiation, are associated with the response to NAC [38, 39]. However, these studies were often single-center, had relatively small sample sizes, and lacked external validation. Some researchers found that tumor T stage was a crucial predictive factor for NAC efficacy [34], similar to what was observed in our study. The rationale behind this may be that T staging reflected tumor burden and invasiveness, with larger or more invasive tumors achieving higher staging before treatment, implying greater resistance or difficulty in treatment. Additionally, higher T stage may indicate greater intratumoral heterogeneity, leading to inconsistent response to chemotherapy drugs [40, 41]. Intratumoral heterogeneity may be reflected, to some extent, by unique textures and spatial grayscale patterns extracted from pre-treatment CT images. However, these theoretical hypotheses have not been fully elucidated through radiogenomic and multiomic studies. Another multicenter study found no significant correlation between clinical-pathologic features and treatment response in LAGC patients [15]. Therefore, the relationship between treatment response in LAGC still requires large-scale studies for validation. Nonetheless, the AUCs of the clinical model exhibited significantly lower values compared to those of the nomogram model (*P* < 0.05). This finding indicates that deep learning models can reveal intricate imaging features and, when combined with clinical features, can more comprehensively quantify intratumoral heterogeneity. Furthermore, enhanced NRI and IDI further corroborated the effectiveness of combining image signatures with clinical features in predicting NAC response.

The proposed nomogram model based on traditional handcrafted, deep learning signatures, and clinical features, in this study can provide a quantitative assessment of patients’ NAC response. The incorporation of radiological signatures related to CT significantly bolstered the predictive capability when added to independent clinical predictive factors. Moreover, it outperformed a single deep learning signature. One possible explanation is that the nomogram model explores high-dimensional imaging features and subsequently quantifies intratumoral heterogeneity comprehensively. In this study, the nomogram incorporated imaging features that are not redundant but complementary. A previous study found that model based on traditional radiomics features could predict the response of LAGC to NAC, but their AUC was 0.77 in the validation set, slightly lower than the nomogram proposed in this study [42]. This might be due to the fact that the previous study did not incorporate a comprehensive analysis of deep learning and clinical features. However, the advantage of that research is that it validated the model’s efficacy in a prospective cohort, achieving favorable results (AUC: 0.72). Another novel study used radiomics nomograms based on MRI to predict the response of LAGC to NAC, with an AUC of about 0.820 in the internal validation set, which is close to this study, but it was a single-center study without external validation [43]. A recent study discovered that extracting deep learning features from the ResNet50 neural network and combining them with clinicopathological features could more accurately predict the response of LAGC to NAC, with AUCs of 0.755 and 0.752 in the internal and external validation sets, respectively. Its efficacy was slightly lower than the nomogram proposed in this study in the internal validation set, possibly because this model did not include manual features [34]. The user-friendly nomogram in this study can be utilized by both clinicians and patients which aligns with the trend of personalized medicine. Additionally, the lowest AIC, along with the enhanced NRI and IDI, also indicate that the improved discriminative capability of the nomogram is attributed to integration rather than overfitting. To evaluate the clinical utility of the nomogram, DCA was conducted. Within a certain threshold range, predicting NAC status using the nomogram provided a net benefit for patients with higher scores. For these patients, alternative treatment strategies with lower toxicity or better tolerance, or close monitoring, can be considered. It is anticipated that the nomogram may ultimately be combined with established clinical-pathologic standards and molecular biomarkers to refine risk stratification and guide personalized management of gastric cancer.

In addition, we investigated the efficacy of the nomogram model for predicting the PFS of patients with LAGC. Our analysis revealed that the nomogram model score independently served as a risk factor for PFS in patients with LAGC following NAC. Patients with lower scores demonstrated better PFS, specifically, those achieving pathologic GR after NAC experienced a significant extension in survival. For gastric cancer, factors influencing the risk of recurrence include a combination of various aspects such as the pre-treatment clinical and pathological characteristics of the tumor, molecular biological features of the tumor, and even the patient’s lifestyle habits [44, 45]. A comprehensive assessment by clinicians is required in clinical practice. For example, patients with larger tumors or higher TNM staging may have a higher risk of recurrence. For patients with lower scores, even those with a high risk of recurrence after surgery, adjuvant chemotherapy may be a suitable option. On the other hand, for patients with higher scores and a lower risk of recurrence, prompt consideration of alternative curative-intent treatment options is warranted to mitigate unnecessary toxicity and enhance survival outcomes. There is still controversy over whether curative resection should be performed after NAC treatment for T2-stage LAGC, or if it should be performed directly without NAC. Some researchers believe that NAC does not prolong recurrence-free and overall survival time after curative resection in T2-stage patients, thus they do not recommend NAC for these patients [46]. The present study may provide guidance on treatment options for such patients through nomogram scoring. In summary, the proposed nomogram in this study may provide a viable approach to facilitate treatment planning. Furthermore, our study identified several independent risk factors for the survival of patients with LAGC, including tumor location, cT staging, and cN staging which are consistent with previous studies [47, 48].

The present study possesses several limitations. Firstly, despite the extensive sample size drawn from multiple centers, inherent biases are unavoidable given the retrospective nature of the study. Patients from different hospitals with varying CT equipment may introduce biases. Therefore, well-designed large-scale studies are imperative to validate the generalizability and clinical applicability of our nomogram model, as prospective validation could further ascertain its predictive accuracy and utility in real-time clinical decision-making. Secondly, different regions or countries may employ NAC protocols and drugs different from those used in this study, which could impact the consistency and generalizability of the study results. Thirdly, tumor delineation was performed on 2D slices rather than the entire tumor in three dimensions, which may not fully represent the entire tumor, and some radiomics features might be influenced by the choice between 2 and 3D analysis. Further research is needed to analyze the entire tumor in three dimensions. In addition, manual segmentation of ROI and manual feature extraction may introduce subjectivity and operational differences, which could affect the accuracy and reproducibility of the results. The application of automated segmentation methods and end-to-end models may help address the aforementioned issues. Fourth, due to the lower pathological complete response rate after NAC in the Chinese population, there is a significant imbalance in the samples. Although oversampling was conducted to compensate for the shortage of positive samples, it led to changes in the data distribution. Fifth, the clear precise biologic significance of the tumor internal features from the deep learning remains unknown and requires comprehensive elucidation. Finally, both this study and several previous studies have only included LAGC patients with postoperative pathological results for evaluating the effectiveness of NAC. However, in clinical practice, some LAGC patients do not undergo surgical resection after NAC, and thus cannot be included in these studies. This may affect the generalizability of the model in the population, particularly for the aforementioned unresectable patients.

This study constructed a personalized prediction model for NAC response in patients with LAGC undergoing surgical resection, integrating deep learning techniques with multi-phase CT images and clinical features. This provided valuable information for clinical practice and processes significant potential for individualized treatment strategies.

## Supplementary Information

Below is the link to the electronic supplementary material.

Supplementary file1 (DOCX 1821 KB)
