# Supplementary material for: Deep learning nomogram for predicting neoadjuvant chemotherapy response in locally advanced gastric cancer patients
Source: Abdom Radiol (NY). 2024 May 26;49(11):3780–96. doi: 10.1007/s00261-024-04331-7 (PMC11519172; doi:10.1007/s00261-024-04331-7)
Supplement: Supplementary file 1 — Supplementary file1 (DOCX 1821 KB) [file 261_2024_4331_MOESM1_ESM.docx]

Supplementary materials

**Supplementary Table S1. The CT protocols of the two centers**

| Parameters | Center A | Center B |
| --- | --- | --- |
| CT version | Brilliance 256-slice iCT scanner (Philips, Netherlands) | GE Revolution 256-slice CT scanner (GE Healthcare, USA)/ SOMATOM Definition Flash dual-source CT scanner (Siemens Healthcare, Germany) |
| CT tube voltage | 120 kVp | 120 kVp |
| CT tube current | 350 mAs | Auto |
| CT rotation time | 0.50 s | 0.60 s |
| CT detector collimation | 128×0.625 mm | 128×0.625 mm |
| Image matrix | 512×512 | 512×512 |
| Field of view | 350×350 mm | 400×400 mm |
| Reconstruction thickness | 1.25 mm | 1.25 mm |
| Contrast agent type | iodoform, Starry Pharm, Shanghai, China | Omnipaque, GE Healthcare, Shanghai, China |
| Contrast agent concentration | 300 mgI/ml | 300 mgI/ml |
| Contrast agent dosage | 1 ml/kg | 1.5 ml/kg |
| Contrast agent infused rate | 3.5 ml/s | 3.0 ml/s |

CT: computed tomography

**Supplementary Table S2. Results of radiomics feature selection and signature building**

| Signatures | Feautres | Coefficients |
| --- | --- | --- |
| Handcrafted signature | Intercept | 0.5 |
|  | Delayed_squareroot_glrlm_RunEntropy | -0.014 |
|  | Venous_logarithm_glszm_SizeZoneNonUniformity | -0.095 |
|  | Venous_log_sigma_5_0_mm_3D_firstorder_RobustMeanAbsoluteDeviation | -0.019 |
|  | Venous_log_sigma_5_0_mm_3D_glcm_Idmn | -0.088 |
|  | Venous_logarithm_glrlm_LongRunEmphasis | -0.017 |
|  | Arterial_wavelet_HHH_glrlm_RunLengthNonUniformityNormalized | -0.023 |
|  | Arterial_wavelet_LHH_glcm_DifferenceVariance | -0.011 |
|  | Delayed_squareroot_firstorder_InterquartileRange | 0.057 |
|  | Venous_log_sigma_1_0_mm_3D_glcm_MCC | -0.048 |
| Deep learning signature | Intercept | 0.5 |
|  | Arterial_Feature_1419 | 0.030 |
|  | Venous_Feature_1211 | -0.004 |
|  | Venous_Feature_678 | -0.010 |

**Supplementary Table S3. Performance of the models**

| Sets | Models | ROC AUC (95% CI) | Acc | Sen | Spe | Pre | P-R AUC (95% CI) | F1_max_ |
| --- | --- | --- | --- | --- | --- | --- | --- | --- |
| Training Set | Handcrafted signature | 0.715(0.650-0.773) | 0.677 | 0.800 | 0.555 | 0.642 | 0.669(0.576-0.750) | 0.754 |
|  | DL signature | 0.809(0.751-0.859) | 0.827 | 0.664 | 0.746 | 0.794 | 0.791(0.705-0.857) | 0.755 |
|  | Clinical model | 0.552(0.484-0.619) | 0.577 | 0.609 | 0.546 | 0.573 | 0.503(0.411-0.596) | 0.667 |
|  | Nomogram model | 0.848(0.793-0.893) | 0.773 | 0.718 | 0.827 | 0.806 | 0.838(0.756-0.895) | 0.782 |
| Internal validation set | Handcrafted signature | 0.559(0.434-0.680) | 0.632 | 0.500 | 0.688 | 0.400 | 0.313(0.151-0.540) | 0.476 |
|  | DL signature | 0.786(0.670-0.877) | 0.721 | 0.950 | 0.625 | 0.514 | 0.518(0.309-0.721) | 0.667 |
|  | Clinical model | 0.629(0.503-0.743) | 0.559 | 0.750 | 0.479 | 0.375 | 0.398(0.212-0.618) | 0.500 |
|  | Nomogram model | 0.802(0.688-0.889) | 0.794 | 0.900 | 0.750 | 0.600 | 0.541(0.329-0.740) | 0.720 |
| External validation set | Handcrafted signature | 0.536(0.432-0.638) | 0.608 | 0.517 | 0.647 | 0.385 | 0.340(0.192-0.526) | 0.4603 |
|  | DL signature | 0.731(0.632-0.816) | 0.763 | 0.552 | 0.853 | 0.615 | 0.542(0.363-0.711) | 0.582 |
|  | Clinical model | 0.588(0.483-0.687) | 0.526 | 0.690 | 0.456 | 0.351 | 0.365(0.212-0.550) | 0.465 |
|  | Nomogram model | 0.751(0.652-0.833) | 0.763 | 0.690 | 0.794 | 0.588 | 0.556(0.376-0.722) | 0.635 |

Acc: accuracy; AUC: Area under the curve; DL: deep learning; Pre: precision; P-R: precision-recall; Sen: sensitivity; Spe: specificity; ROC: receiver operator curve;

**Supplementary Table S4. Difference between areas when handcrafted signature and clinical model compared with deep learning signature**

| Sets | Model | Difference between areas | 95% Bootstrap CI ^a^ |
| --- | --- | --- | --- |
| Training set | handcrafted signature | 0.122 | 0.097 to 0.155 |
|  | clinical model | 0.288 | 0.241 to 0.323 |
| Internal validation set | handcrafted signature | 0.205 | 0.117 to 0.351 |
|  | clinical model | 0.120 | 0.019 to 0.246 |
| External validation set | handcrafted signature | 0.203 | 0.144 to 0.287 |
|  | clinical model | 0.178 | 0.075 to 0.274 |

**Note:** ^a^ bootstrap confidence interval (1000 iterations; random number seed: 978). The 95% Bootstrap CI of all difference between areas does not contain 0.

**Supplementary Table S5. Related factors for GR detection in LAGC**

| Intercept and variable | Odds ratio (95% CI) | P |
| --- | --- | --- |
| Intercept | -- | <0.001 |
| Clinical T stages  T2+3  T4a+4b | Ref  2.793 (1.367-5.703) | 0.005 |
| Handcrafted signature | 2.168 (1.462-3.215) | <0.001 |
| Deep learning signature | 4.049 (2.597-6.313) | <0.001 |

**Supplementary Table S6. NRI and IDI between the nomogram and clinical model in training, internal validation, and external validation sets**

|  | Training set | P | Internal validation set | P |  | External validation set | P |
| --- | --- | --- | --- | --- | --- | --- | --- |
| Nomogram vs. Clinical |  |  |  |  |  |  |  |
| NRI | 0.295(0.212-0.375) | <0.001 | 0.173(0.043-0.311) | 0.011 |  | 0.163(0.014-0.318) | 0.038 |
| IDI | 0.591(0.424-0.749) | <0.001 | 0.347(0.087-0.621) | 0.011 |  | 0.326(0.029-0.636) | 0.038 |

IDI: Integrated Discrimination Improvement; NRI: Net Reclassification Improvement.


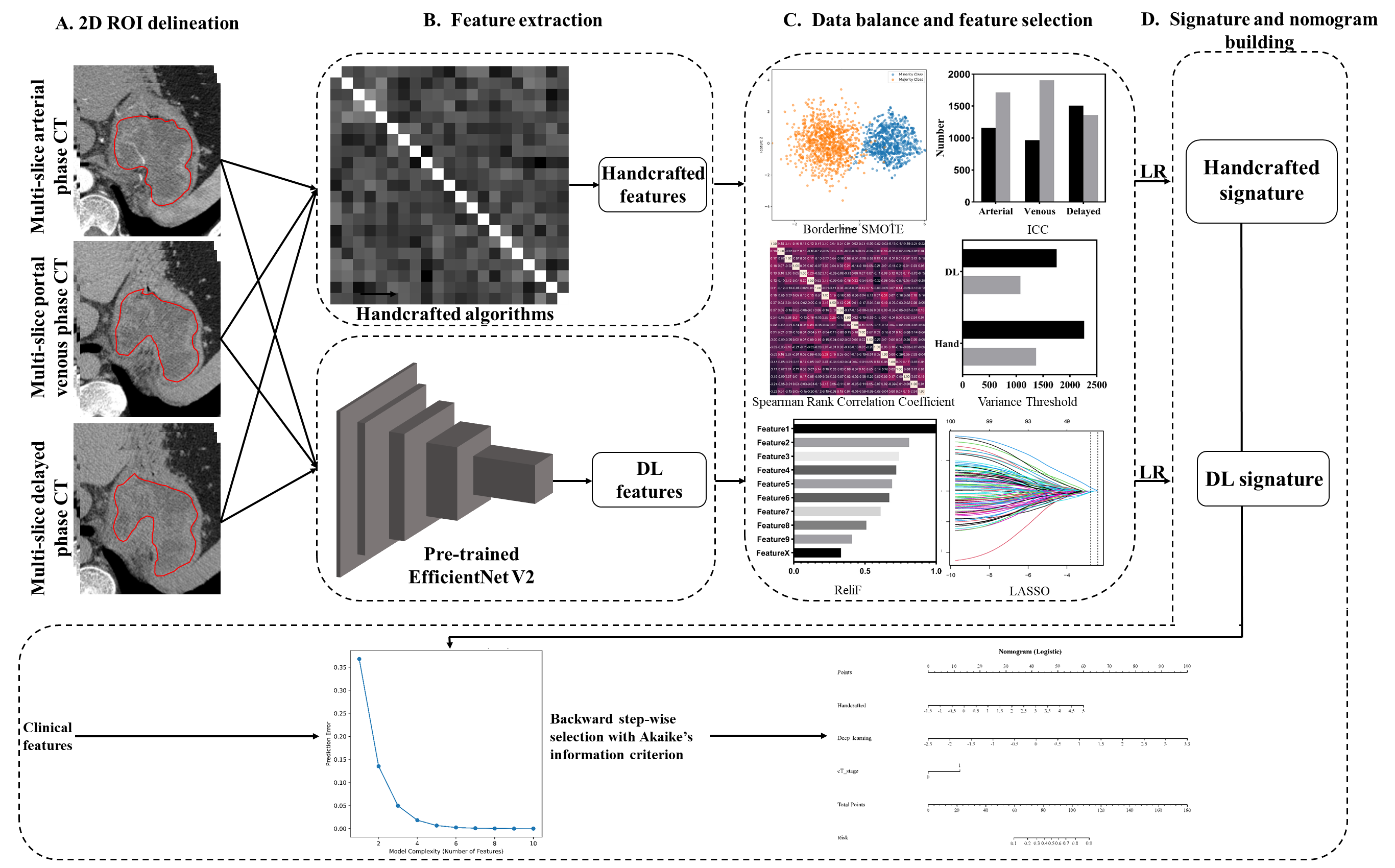


**Supplementary Figure S1. Workflow of the study.** (A) Delineation of two-dimensional region of interest (2D ROI) was conducted on multi-slice arterial, portal venous, and delayed-phase computed tomography (CT) images. The delineation included areas of tumor necrosis and hemorrhage, while ensuring avoidance of gastric gas and fluids. (B) Feature extraction was performed using handcrafted algorithms and a pre-trained EfficientNet V2 convolutional neural network (CNN) to respectively obtain handcrafted features and deep learning (DL) features from contrast-enhanced images in three phases. (C) Borderline synthetic minority over-sampling technique (Borderline SMOTE) was utilized to resample minority samples until the number of positive and negative samples was equal; feature selection was performed stepwise using interclass/intraclass correlation coefficient (ICC), Spearman rank correlation coefficient, variance threshold, ReliefF, and least absolute shrinkage and selection operator (LASSO) regression. (D) Logistic regression was employed to establish models using both handcrafted features and DL features. Combining logistic regression with backward step-wise selection using Akaike’s information criterion to construct a nomogram.


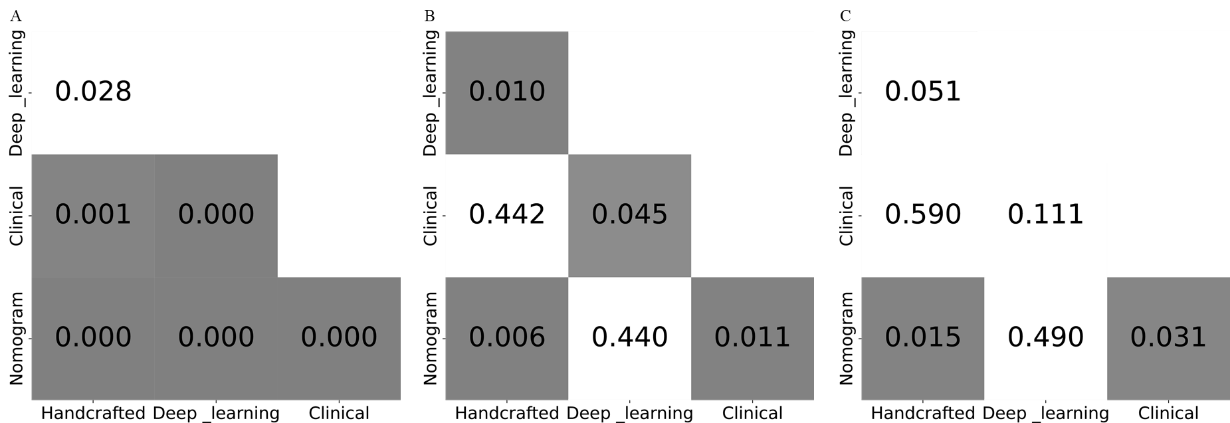


**Supplementary Figure S2. Comparison results of receiver operator curves, where the values in the squares represent the P-values of the DeLong test.** (A-C) correspond to the comparison results of the training set, internal validation set, and external validation set, respectively.


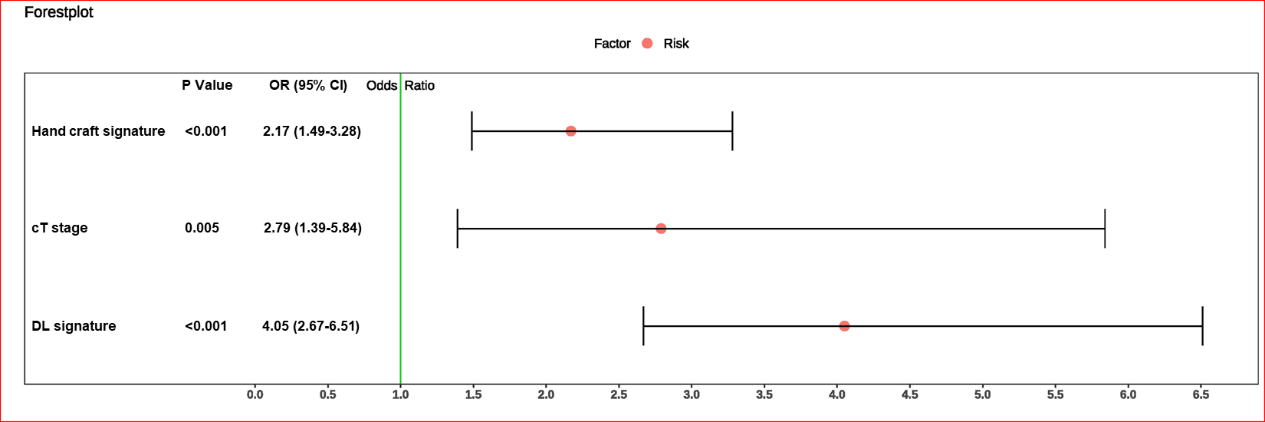


**Supplementary Figure S3. Forest plot of the nomogram in the training set.**

95% CI: 95% confidence interval: DL: deep learning.
